# Case Report: Cutaneous metastatic mucin-producing prostate adenocarcinoma

**DOI:** 10.3389/fonc.2025.1681839

**Published:** 2025-11-10

**Authors:** Ahmet M. Yildirim, Douglas Semler, Jeffrey Harvell, Eun-Mi Yu, Sekwon Jang, Suraj Venna

**Affiliations:** 1 Department of Internal Medicine, Inova Fairfax Hospital, Falls Church, VA, United States; 2 Department of Dermatology, Semler Dermatology Inc., Landsdowne, VA, United States; 3 Department of Pathology, Inova Fairfax Hospital, Falls Church, VA, United States; 4 Genitourinary (GU) Medical Oncology, Inova Schar Cancer Institute, Fairfax, VA, United States; 5 Inova Melanoma and Skin Cancer Center, Inova Schar Cancer Institute, Fairfax, VA, United States

**Keywords:** prostate adenocarcinoma, mucin-producing, cutaneous, BPH, PSA, PSAP, PIN4, NKXC3

## Abstract

Cutaneous metastasis of prostate adenocarcinoma is exceedingly rare, accounting for less than 1% of all skin metastases. It typically occurs late in the disease course and often mimics benign or primary skin malignancies. An elderly man with a history of benign prostatic hypertrophy presented for a routine dermatologic examination, during which an ulcerated nodule was identified on his upper back. Histopathology of the lesion revealed a dermal-based malignant glandular tumor. Immunohistochemistry was negative for CK20, CK5/6, PAX-8, TTF-1, PSA, and PIN4, but positive for PSAP and NKX3.1, consistent with mucin-producing prostate adenocarcinoma. Although his recent digital rectal exam was normal and prostate cancer screening had been deferred due to age, further work-up showed elevated serum PSA (30.49 ng/mL) and widespread metastatic disease on PET/CT imaging. He was started on androgen deprivation therapy with relugolix. This case underscores the diagnostic importance of skin biopsies in evaluating atypical lesions and highlights a rare presentation of metastatic prostate adenocarcinoma in an elderly patient with minimal systemic symptoms. It also raises awareness of prostate cancer presenting as solitary cutaneous metastasis and calls attention to the potential value of PSA screening in select elderly patients. Recognizing uncommon cutaneous manifestations may lead to earlier diagnosis and improved management of advanced internal malignancies.

## Introduction

Prostate cancer is the most common cancer in men in the United States (excluding skin cancer) and the second-leading cause of cancer-related death following lung cancer (1). Patients with advanced prostatic adenocarcinoma (PAC) typically present with genitourinary or musculoskeletal symptoms due to tumor burden in the pelvis and bones. PAC is associated with distinct immunologic profiles (2). Metastatic PAC to the skin is rare and can present as an ulcerated skin nodule, typically on the trunk. We report a case of a metastatic mucin-producing PAC initially presenting as an ulcerated skin nodule.

## Case report

**Figure 1:**
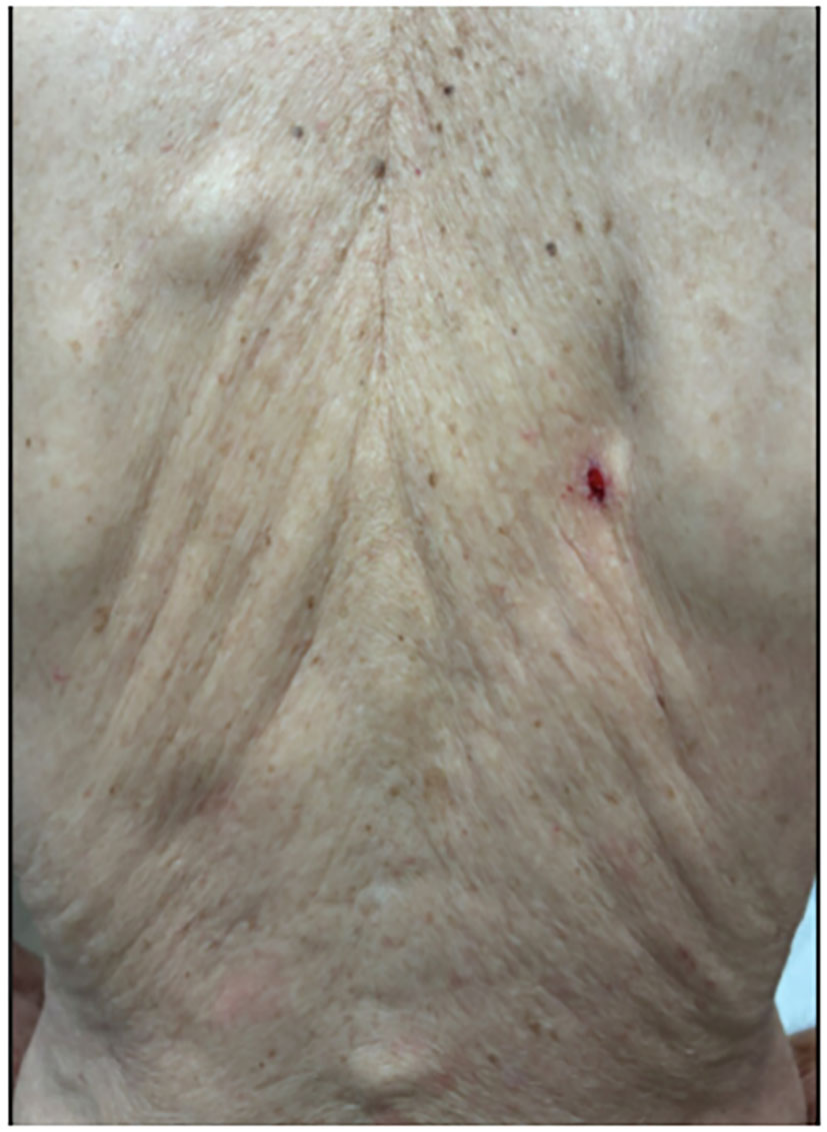
One-centimeter ulcerated nodule on the patient’s mid-back.

**Figure 2:**
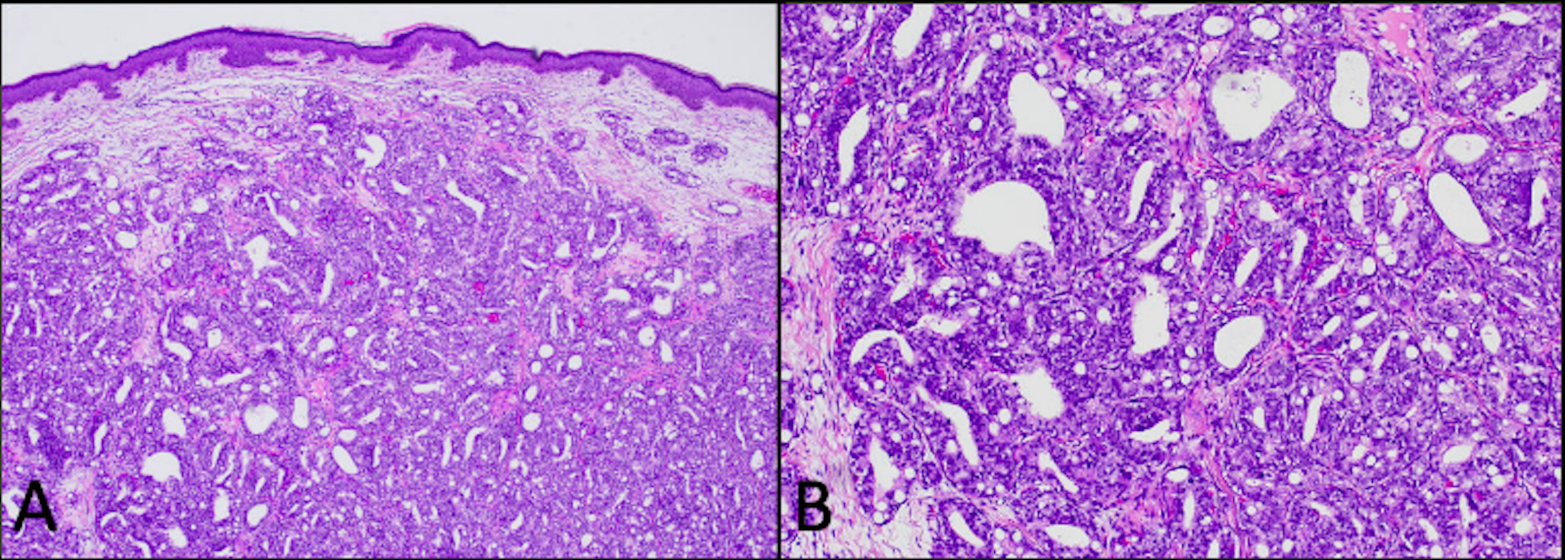
Hematoxylin and eosin (H&E)-stained punch biopsy of the edge of the ulcer revealed a dermal-based malignant glandular tumor [**(A)** 4×] with mucin production [**(B)** 100×].

**Figure 3:**
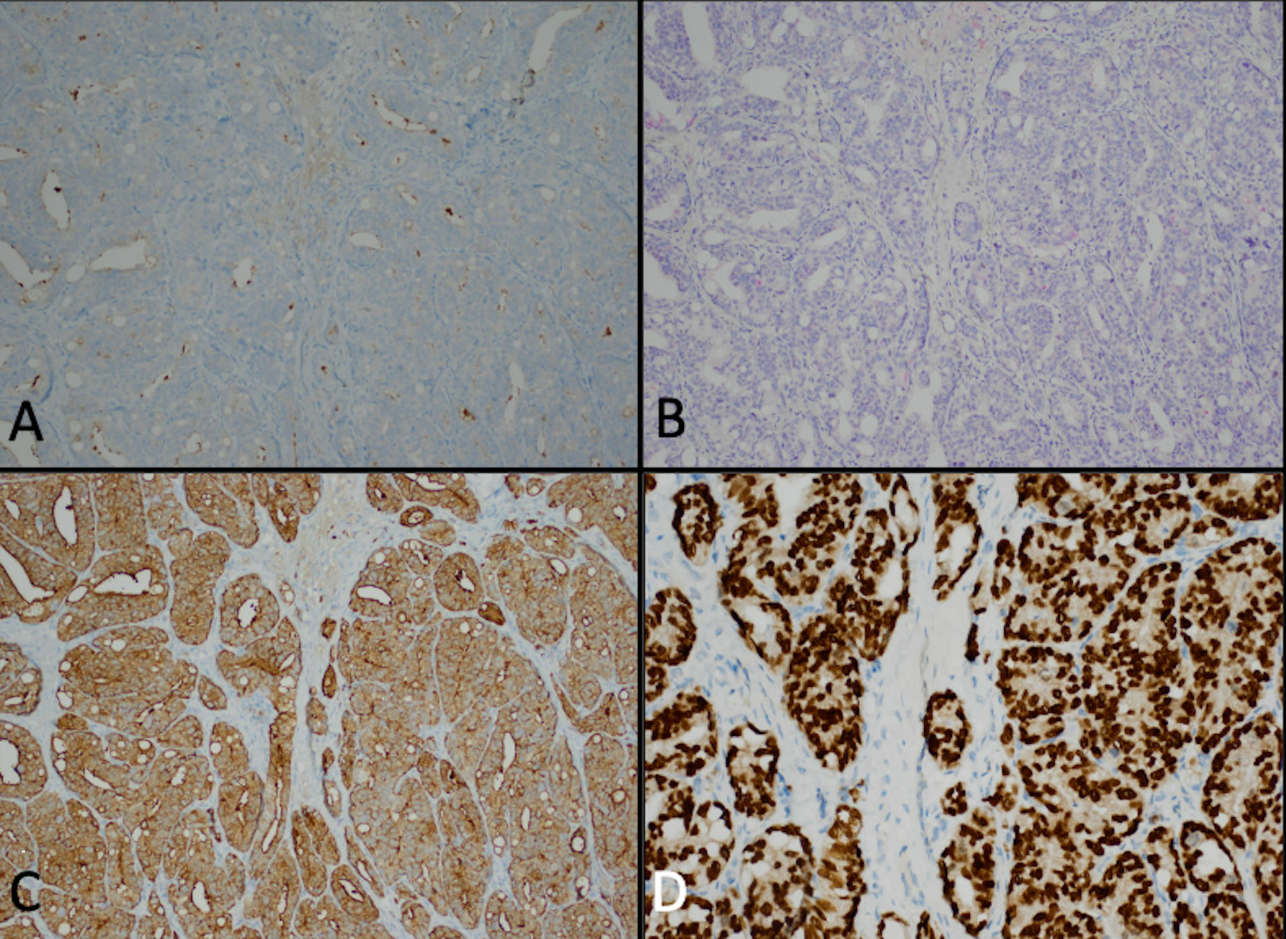
Punch biopsy of the edge of the ulcer revealed weak staining for PSA **(A)** and PIN4 **(B)** as well as strong expression of PSAP **(C)** and NKXC3 **(D)**.

A 91-year-old white man with a history of benign prostatic hypertrophy (BPH) presented to his dermatologist for a skin examination for an unrelated complaint. On exam, an ulcerated pink-to-skin-colored nodule was noted on the right upper back (**Figure 1**). Histology from a punch biopsy from the edge of the nodule revealed a dermal-based malignant glandular tumor (**Figure 2**). Immunohistochemistry (IHC) was negative for CK20, CK5/6, PAX-8, and TTF-1, as well as the prostate specific markers, prostate-specific antigen (PSA) and PIN4. Lesional cells were positive for the prostate markers PSAP and NKXC3, consistent with a mucin-producing adenocarcinoma (**Figure 3**). Two months earlier, he had undergone a digital rectal exam (DRE), which was reported as normal. Based on his age, prostate cancer screening with a PSA test was initially deferred, and he resumed tamsulosin for his BPH. Metastatic workup included a whole-body positron emission tomography/computed tomography (PET/CT) scan, which showed multiple hypermetabolic lesions, including the left scapula, axial spine, mediastinal lymph nodes, and an enlarged prostate. Serum PSA level was obtained and found to be elevated to 30.49 ng/mL. These findings were compatible with a metastatic, mucin-producing PAC. The patient began androgen deprivation therapy with relugolix, a GnRH antagonist, with plans to add on an androgen receptor pathway inhibitor (ARPI) with the primary goal of symptom palliation and preservation of his quality of life given his advanced age.

## Discussion

In this case, we report a 91-year-old white man with BPH who presented for a routine skin examination and found to have an ulcerated skin nodule that led to the discovery of widely metastatic prostate cancer. The IHC pattern from the skin nodule was compatible with an aggressive subtype of PAC with loss of expression of the prostate markers PSA and PIN4, along with the retention of prostate markers PSAP and NKXC3.

Generally, internal malignancies will involve the skin in 2%–9% of cases (3, 4), and prostate cancers account for less than 1% of all skin metastases (5). Cutaneous metastases from prostate cancer typically involve the lower abdomen, genitals, and thighs (6), likely due to the proximity to the prostate gland via hematogenous dissemination. Additionally, previous case reports indicate that when cutaneous manifestations are present, they are often diagnosed late in the disease course after systemic symptoms or other metastatic sites have appeared (7, 8). This delay may be related to rarity and nonspecific presentations, which may further lead to misdiagnosis or deferred biopsy.

In our patient’s case, his urinary frequency and retention may have been the first symptoms indicative of PAC in spite of a normal DRE 2 months prior. Notably, he did not present with symptoms of widespread metastases disease such as bone pain, weight loss, fatigue, hematuria, constipation, or dry cough. The differential diagnoses of his skin lesion included primary skin cancers such as basal cell carcinoma, squamous cell carcinoma, Merkel cell carcinoma, and amelanotic melanoma. This case highlights the importance of a biopsy for a definitive diagnosis, as it represents a unique presentation of a cutaneous metastases arising from another solid primary malignancy. It also highlights the potential impact of prostate cancer screening in minimally symptomatic elderly patients on early diagnosis and management.

The pathways of prostate cancer metastasis to the skin are not well-understood due to its rarity. Possible routes include lymphatic or hematogenous spread for distant disease and direct extension to the skin from an underlying prostate cancer (9). It has been theorized that serine proteases, specifically human tissue kallikreins, may facilitate dermal extravasation by disrupting epidermal adhesions (10). We surmise that in our patient’s case, he developed spine and dermal metastases via hematogenous spread, as his fluorodeoxyglucose (FDG) PET/CT scan did not reveal any other metastatic lesions that would have extended directly to the dermis of his right mid-back.

Patients with widespread metastatic prostate cancer typically present with pain or systemic symptoms like fatigue or weight loss. In some instances, metastatic prostate cancer is diagnosed after routine PSA screening. Our case is notable for a solitary cutaneous lesion in a patient with no prior history of prostate cancer. In contrast, another reported case of prostate cancer skin metastases was a 67-year-old man with a 4-year history of castration-resistant prostate cancer who developed extensive cutaneous involvement on the lower abdomen, inguinal area, and left flank areas. Despite aggressive treatments, including surgery, chemotherapy, and androgen deprivation therapy, he was ultimately referred to hospice care (7). The 5-year survival of advanced prostate cancer is approximately 30% (11), consistent with the disease course observed in the aforementioned case. Both of these cases underscore the reality that advanced prostate cancer, like other malignancies, can manifest in unusual and diverse ways. A more comprehensive understanding of the variable presentations of cutaneous manifestation in prostate carcinomas is warranted.

## Conclusion

This case highlights the importance of including cutaneous metastasis from solid organ malignancies in the differential diagnosis of atypical skin lesions, particularly in the elderly.

## Data Availability

The raw data supporting the conclusions of this article will be made available by the authors, without undue reservation.
